# Sex-Bias in Irritable Bowel Syndrome: Linking Steroids to the Gut-Brain Axis

**DOI:** 10.3389/fendo.2021.684096

**Published:** 2021-05-19

**Authors:** Sik Yu So, Tor C. Savidge

**Affiliations:** ^1^ Department of Pathology and Immunology, Baylor College of Medicine, Houston, TX, United States; ^2^ Texas Children’s Microbiome Center, Department of Pathology, Texas Children’s Hospital, Houston, TX, United States

**Keywords:** sex steroids, irritable bowel syndrome, gut-brain axis, gut microbiota, androgens, estrogens

## Abstract

Irritable bowel syndrome (IBS) is a functional gastrointestinal disorder that is more common in females. Despite its high global incidence, the disease mechanism is still unclear and therapeutic options remain limited. The sexual dimorphism in IBS incidence suggests that sex steroids play a role in disease onset and symptoms severity. This review considers sex steroids and their involvement in IBS symptoms and the underlying disease mechanisms. Estrogens and androgens play important regulatory roles in IBS symptomology, including visceral sensitivity, gut motility and psychological conditions, possibly through modulating the gut-brain axis. Steroids are regulators of hypothalamic-pituitary-adrenal activity and autonomic nervous system function. They also modulate gut microbiota and enteric nervous systems, impacting serotonin and mast cell signaling. Sex steroids also facilitate bidirectional cross-talk between the microbiota and host following bacterial transformation and recycling of steroids by the intestine. The sex-specific interplay between sex steroids and the host provides neuroendocrinology insight into the pathophysiology, epigenetics and treatment of IBS patients.

## Introduction

Irritable bowel syndrome (IBS) is a common functional gastrointestinal (FGID) disorder that is characterized by abdominal pain and disturbances in bowel habit. It is classified according to predominant stool patterning into several subtypes, which include IBS with constipation (IBS-C); IBS with diarrhea (IBS-D); IBS with mixed stooling patterns (IBS-M); and unsubtyped IBS (IBS-U). Irrespectively of bowel habit, IBS is diagnosed based on symptoms experienced, notably using Rome definitions that were recently updated to Rome IV criteria (1). The global prevalence of IBS is estimated at 9.2% versus 3.8% based on diagnosis by Rome III and Rome IV criteria, respectively (2). Not only do IBS patients suffer from abdominal discomfort, but their quality of life is also impacted by extra-intestinal symptoms including anxiety, depression, headache and fatigue (3, 4). Major limitations in the field include uncertainty regarding IBS disease etiology and limited therapeutic options that show efficacy in subpopulations of patients only. Notably, females are more frequently diagnosed with IBS and clinical symptoms tend to be more severe (2, 5). The sex-bias in IBS was recently reviewed (6–8), suggesting that sexual dimorphism and sex steroids could be involved in the pathophysiology, a view supported by symptoms being linked to estrous cycle (9) and the absence of X-linked genetic susceptibility (10). Building on this previous body of work, here we provide a deeper dive into sex steroids and their involvement in IBS symptoms, focusing on their molecular mechanism of action and impact on the microbiome-gut-brain axis.

## Current Evidence on Sex and IBS Incidence

**Figure 1:**
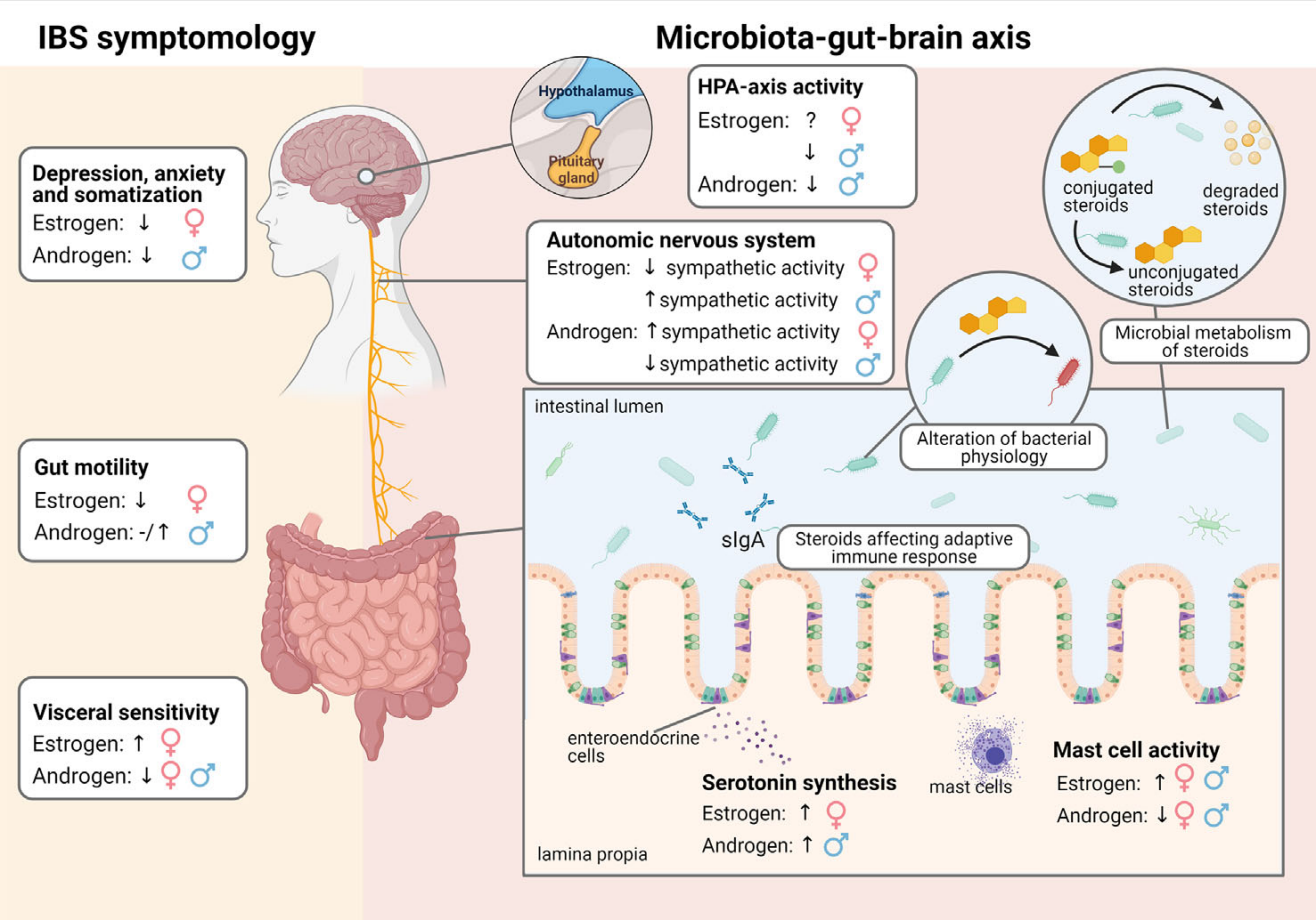
Effect of estrogens and androgens on IBS symptomology and the microbiota-gut-brain axis in males and females.

IBS is more common in females (2, 11). This trend was also found in children (12), although some inconsistent results have been reported (13). While IBS prevalence is constant with age in males (14), disease prevalence in females is age and hormonal status dependent. Some inconsistencies are reported, for example a few notable studies report a reduction in IBS incidence after menopause, whereas others show exacerbated FGID symptoms (15, 16). Sex is also a determinant of IBS subtype, with constipation being predominant in females, whereas diarrhea is more frequently diagnosed in males (17, 11). Females are also more likely to suffer from more severe clinical symptoms (5), although other studies did not report a sex-bias in disease severity. A number of confounding factors such as cultural differences could contribute to this discrepancy. For example, in India males are diagnosed with IBS more frequently than females, but this may be linked to males being more likely to consult a physician (18). Conversely, in Europe and North America, physician IBS consultations are reported as female-biased (11). Nevertheless, the literature generally supports sexual dimorphism in IBS and suggests that sex-related physiological differences, such as sex steroids could be related to disease onset and symptoms severity (**Figure 1**).

## Sex Steroids

**Figure 2:**
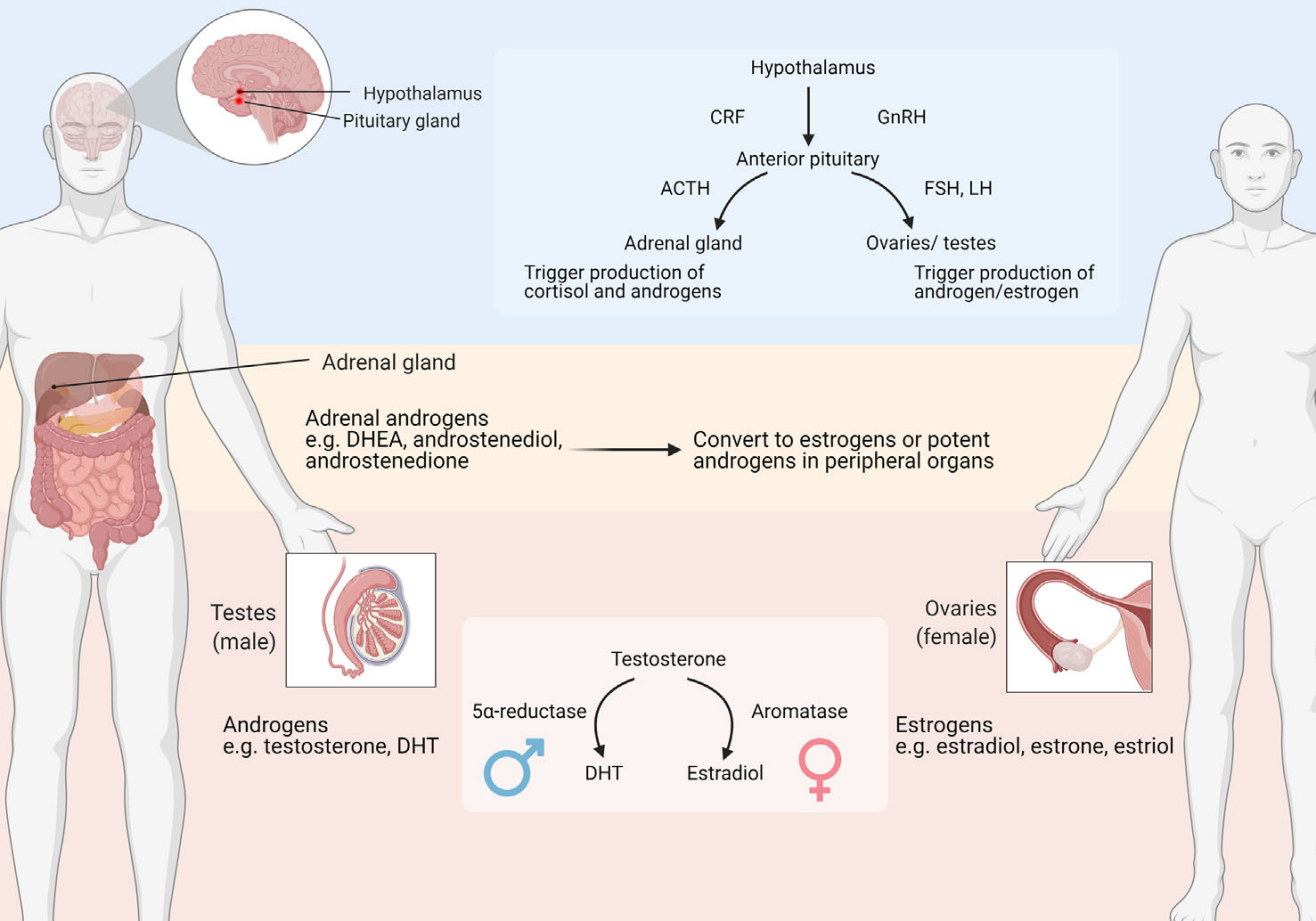
Major organs involved in sex steroids production. ACTH: adrenocorticotropic hormone; CRF: corticotropin-releasing factor; DHEA, dehydroepiandrosterone; DHT, Dihydrotestosterone; FSH, Follicle-stimulating hormone; GnRH, Gonadotropin-releasing hormone; LH, Luteinizing hormone. Adapted from “Primary and Secondary Endocrine Organs” by BioRender.com (2021). Retrieved from https://app.biorender.com/biorender-templates.

Sex steroids play an important role in reproductive system homeostasis, as well as in other physiological processes. There are three main types of sex steroids: namely estrogens, androgens and progesterone. Among these, this review focuses mainly on estrogens and androgens. Estrogens are mainly produced in the ovaries in premenopausal women, whereas the testis or other peripheral organs are involved in hormone production in men (**Figure 2**). The major forms of estrogens are estradiol, estrone and estriol. The estrogen forms exhibit varied potencies and play different roles during development. For instance, estradiol is the most abundant and potent estrogen during female reproductive years, while estrone predominates after menopause (19). Estrogens bind to and activate classical estrogen receptors (ER), including receptor α (ERα) and β (ERβ) isoforms which belong to the family of nuclear hormone receptors (NHRs) that translocate to the nucleus where they bind to DNA and mediate the genomic effects of estrogens (20). Estrogens also bind to membrane-bound receptors, such as G-protein-coupled estrogen receptor (GPER) to exert non-genomic effects. Upon binding and activation, GPER triggers downstream pathways and regulates a wide range of activities, including cellular differentiation and proliferation.

Androgenic steroids mainly include testosterone and dihydrotestosterone (DHT). Androgens are mostly produced by the testis in males, and are derived from the ovaries or adrenal glands in females (**Figure 2**). Androgens bind to and activate the androgenic receptor (AR), another member of NHRs. Similar to estrogens, androgens also activate membrane-associated receptors, including G protein-coupled receptor class C group 6 member A (GPRC6A) and the zinc transporter ZIP9 (21). Androgens can be converted into estrogen, through an enzymatic process catalyzed by aromatase that occurs preferentially in females (**Figure 2**).

The adrenal glands produce various adrenal C19 steroids, also known as the adrenal androgens, including dehydroepiandrosterone (DHEA), androstenediol, androstenedione and 11β-hydroxyandrostenedione (22). These steroids are usually weaker ligands of androgen and estrogen receptors, but they represent significant circulating precursors for peripheral conversion to potent androgen and estrogen forms. After release from the adrenal cortex, adrenal steroids are converted to testosterone or DHT through enzymatic reactions that contribute substantially to circulating testosterone levels in pre-pubertal children and women. Furthermore, the 11-oxygenated androgen 11β-hydroxyandrostenedione is released in significant quantities by the adrenal glands, and contributes to the synthesis of 11-keto-testosterone and 11-keto-dihydrotestosterone which have similar androgen receptor activation potential compared to testosterone and DHT (23–25). These studies demonstrate that adrenal androgens are also important when studying the potential role of sex steroids in IBS patients.

Sex steroids play a critical role in many processes including gender development, skeletal growth, brain function, etc. They are also associated with various diseases, including obesity, Alzheimer’s disease, autism in males and females. For instance, testosterone deficiency is associated with obesity and insulin resistance in males (26); while excessive testosterone is associated with these traits in females (27). The decline in sex hormone levels with advancing age also causes undesirable aging-associated outcomes, for example reduced estrogen around menopause leads to menopausal symptoms and osteoporosis in females. Despite the low androgenic potency, adrenal C19 steroids are considered neuroactive due to their ability to modulate neuroreceptors such as GABA A receptors (28). These steroids play an important regulatory role in various functions, for example cognition, anxiety and depression. Detailed physiological studies that investigate how sex steroids are associated with IBS disease onset and symptoms are therefore warranted.

## Association of Sex Steroids With IBS Symptoms and Treatment

The female predominance of IBS indicates that ovarian hormones, particularly estrogens could act as disease modulating signals. For example, a recent study reported significantly higher serum estradiol in females with IBS than healthy controls (29). While most IBS-related studies have focused on estrogen and ovarian hormones, several studies have also investigated the role of androgens in disease incidence. Males with IBS exhibited less male characteristics when assessed using a male-trait score (30), demonstrating a potential role for androgens. However, a study found higher testosterone in male IBS patients compared to healthy controls (31). In females, patients with polycystic ovary syndrome have higher androgen levels and IBS risk than other females (32). A more systematic evaluation of sex steroid levels in IBS, paying careful attention to age and methodological variables is therefore needed.

### Visceral Hypersensitivity

One key clinical manifestation of IBS is visceral hypersensitivity, which refers to the heightened sensation to physiological stimuli in visceral organs. Females generally demonstrate higher sensitivity to pain (33–35). Yet, a recent report questioned this generalization after finding no sex differences in visceral sensitivity in young healthy participants (36). Animal studies show that sex steroids, in particular estrogen, are important regulators of visceral hypersensitivity and contribute mechanistically in a number of ways. Estrogens are generally reported to increase visceral sensitivity (37–39). However, sex and concentration of estrogens may affect their mechanisms of action. For example, one study reported that administration of high levels of estradiol is analgesic only in female but not male rats (40). Expression and activation of estrogen receptors also appears to be important, with ERα and GPER receptor expression being upregulated and correlated positively with gut pain in patients with IBS-D (41). Estrogen receptors are distributed along the entire visceral pain sensation pathway in rodents (42–44). It is therefore reasonable to assume that estrogens modulate visceral sensitivity at both peripheral and central nervous system levels. In the periphery, estrogens modulate nociceptive responses through altering ion channel opening, G-protein coupled signaling and nociceptive receptor expression (45). Among the ion channels, the transient receptor potential vanilloid-1 (TRPV1) channel is involved in thermal and pain sensation and is well recognized to play an important role in visceral hypersensitivity. TRPV1-expressing sensory fibers are increased in colonic tissue from IBS patients and are positively correlated with abdominal pain score (46), reflecting the potential involvement of TRPV1 in IBS. After activation, the entry of calcium ions transduce the release of neuropeptides or excitatory compounds which activate pain transmission pathways and eventually lead to visceral pain and IBS symptoms (47). Estrogens sensitize TRPV1 and upregulate TRPV1 expression in sensory neurons in rodents and *in vitro* studies (48–50). This response could contribute to the sexual dimorphism of pain perception. Estrogens can modulate TRPV1 expression *via* genomic or non-genomic regulatory pathways. For genomic action, estrogens bind to ER, which translocates into the nucleus, binds DNA estrogen response elements (ERE) in the promoter region of the TRPV1 gene and upregulates transcription of TRPV1 (51). Other mechanisms can also promote estrogen modulation of hypersensitivity, including regulation of opioid receptors (52).

In contrast to estrogens, androgens are usually reported as antinociceptive. In rats, estradiol increases stress-induced visceral hypersensitivity in males, while testosterone reduces it in females (38). In a study of male rats, testosterone negatively correlated with rectal sensory threshold to balloon distension (53), suggesting a protective role of androgens. However, other studies reported that testosterone does not influence visceral pain in either male or female animal models (40). Androgens also modulate the TRP family, including TRPV1, but transient receptor potential melastatin 8 (TRPM8) represents the main target of androgens (54, 55). TRPM8 is a receptor involved in cold perception and treatment with DHT elevates TRPM8 expression *in vitro* (56). Compared with TRPV1, the involvement of TRPM8 in IBS and pain perception is less clear and needs further study. A report investigating TRPM8 polymorphism found an association with increased IBS risk (57). While TRPM8 is suggested to possess both pro- and anti-nociceptive roles in the intestine (58), ligands of TRPM8 such as peppermint are believed to possess analgesic effects in IBS patients (59, 60). Mechanistically, these anti-nociceptive properties could be mediated through activation of TRPM8 on peripheral sensory neurons, which subsequently desensitizes TRPV1 activation (58). Altogether, androgens possibly could reduce visceral pain through enhancing TRPM8 expression and/or activity.

### Gastrointestinal Dysmotility

Another key clinical manifestation of IBS is altered gastrointestinal motility, which also defines IBS subtypes. In general, intestinal transit is slower in women than in men (61) (62–64), and could contribute to the predominance of IBS-C in females versus IBS-D in males. Estrogens delay intestinal motility, possibly through modulation of the nitric oxide (NO)/cyclic guanosine monophosphate (cGMP) pathway *via* ER and GPER activity. Sex-related differences in gut motility could also relate to higher ERα and ERβ expression in females (65). Elevated ER expression in females is linked to increased responses to estrogens with stimulation of nitric oxide (NO) and cGMP secondary messengers, resulting in activation of smooth muscle relaxation. Similar to ER, GPER-coupling inhibits intestinal motility by stimulating NO release (66). GPER agonist G1 and estradiol prolong colonic transit times in both male and female mice and inhibit colonic muscle contraction *in vitro* (67). Also, administration of GPER antagonist G15 reduces colonic transit time and inhibits the effect of estradiol in female mice (66). Even though GPER mRNA levels are reported to be higher in IBS-D compared with IBS-C patients or healthy individuals (68, 41), the consensus opinion favors involvement of GPER in alteration of bowel movement in IBS. In addition to estrogen receptors, TRPV1 may also play a role in motility disorders (69), although the results have been conflicting. A study reported that activation of TRPV1 causes neuronal release of tachykinins which mediate an increase in gut motility (70); whereas another study showed inhibition of jejunal motility by TRPV1 through the NO signaling pathway (71). The different doses of agonist administered could represent a confounding factor (72). Little evidence is available to understand the regulatory role of androgens on gastrointestinal motility. The impact of testosterone ranges from showing no influence on intestinal motility in male rats (73), to more recent studies reporting that androgens induce intestinal smooth muscle contraction through a non-genomic calcium sensitization pathway in intestinal tissues from male rodents (74, 75). Further investigations are required to confirm whether and how androgens influence gut motility.

### Psychological Symptoms

Apart from gastrointestinal discomfort, IBS patients often suffer from psychological symptoms, including depression, anxiety and somatization (**Figure 1**). Women are more commonly affected by these symptoms than men, in both IBS patients (76–78) and healthy individuals (79–81). Low levels of hormones are closely associated with these psychological symptoms and fluctuations in ovarian hormones contribute to mood-related symptoms in females. For instance, depression and anxiety are common symptoms of premenstrual syndrome (PMS), which occurs when estrogen and progesterone levels decrease during the menstrual cycle (82). Estrogen receptors however exert divergent effects, which could contribute to the inconsistent effects reported for estrogen treatment on anxiety (83). Meanwhile, androgen deficiency and AR dysfunction contribute to anxiety and depression in male rodents, whereas testosterone therapy alleviates the symptoms in men (84, 85). Moreover, the adrenal androgen DHEA exhibits antidepressant properties and is reduced during depression (86, 87).

### Relationship Between Estrous Cycle and Gastrointestinal Symptomatology in IBS

The female predominance of IBS highlights the potential role of ovarian steroids in the pathophysiology. Fluctuations of gastrointestinal symptoms during the estrous cycle demonstrate the importance of ovarian steroids. The estrous cycle is mainly divided into menstrual phase, follicular phase, ovulation and luteal phase. Estrogens increase during the follicular phase, and drop rapidly at ovulation. Levels of estrogen and progesterone then increase gradually and dominate during the early luteal phase. Women have prolonged gastrointestinal transit times at this stage compared to the follicular phase (64, 88). During the perimenstrual phase when ovarian hormones are generally at their lowest levels, women experience enhanced visceral sensitivity and gut pain (89). Furthermore, IBS patients often experience enhanced symptoms during menses (90). Houghton et al. 2002 reported that rectal sensitivity was increased during menses in IBS patients but not in healthy volunteers (91). Other examples demonstrating the effect of sex steroids on gastrointestinal function include hormone therapy and pregnancy which are suggested to influence gut motility and visceral sensitivity (92–94). How sex steroids impact motility and psychological symptoms in IBS is likely closely related to gut-brain-axis signaling and include mechanistic components such as serotonin function and the HPA axis which are considered in the next section.

### Therapeutic Options

Sex-dependent efficacy of currently available IBS treatments has been reported in various studies and was reviewed recently (95). Males and females are found to respond differently to some treatments. For example, alosetron which targets the serotonergic pathway is more effective in females, possibly due to sex differences in drug metabolism and receptor-signaling (*discussed in later section*) (96, 97). However, there are limited studies that directly evaluate how sex steroids affect the action of IBS treatments. When considering their close association with IBS symptomology, it is possible that sex steroids modulate treatment efficacy. For example, peppermint oil efficacy against IBS contains menthol, a TRPM8 activator, as the bioactive ingredient (98). As androgens also target TRPM8, it could influence the efficacy of peppermint oil through ion channel competition. It is therefore important to evaluate how sex steroids may influence and predict treatment outcomes.

## Sex Steroids and the Gut-Brain Axis in IBS

One of the major disease mechanisms proposed in IBS is gut-brain axis dysregulation. Neuroimaging studies demonstrate that patients with IBS have different brain morphology when compared with healthy individuals (99, 100), suggesting significant contributions of altered brain circuits in IBS development. Researchers have focused on several components of the gut-brain axis in IBS. Stress is one of the risk factors associated with onset and exacerbation of IBS. For instance, a recent study reported that stress during the COVID-19 pandemic exacerbated clinical symptoms in IBS patients (101). Stress response mechanisms could therefore play an important regulatory role in IBS pathophysiology.

### Hypothalamic-Pituitary-Adrenal (HPA) Axis

IBS patients are more likely to have been exposed to stress as early adverse life events (102–105), which is often related to dysregulation of the HPA axis (**Figure 1**). Among IBS patients, those who experienced childhood trauma are more likely to suffer from somatization and psychological distress (106). The HPA axis is an important component of the neuroendocrine system and plays a pivotal role in mediating the stress response. In response to stress, corticotropin-releasing factor (CRF) is released from the hypothalamus. This results in the release of adrenocorticotropic hormone (ACTH) from the pituitary gland, which causes the adrenal cortex to release glucocorticoid hormones, such as cortisol, which in turn downregulates the pathway as a negative feedback loop (107). The HPA axis is related to various stress responses, including visceral sensitivity and depression, and is dysregulated in IBS (108), although the exact association between IBS symptoms and the HPA response is not consistently reported. In some studies the HPA axis response is enhanced in IBS patients compared with controls (109–111), while another study reported the opposite finding (112). Yet another study reported that the HPA response in IBS patients was enhanced in males but blunted in females (113), demonstrating the importance of considering sex-bias when considering the stress response in IBS.

HPA axis activation related to sex-bias is also controversial (113, 114). Different hormonal status such as estrous cycle, menopause and pregnancy are known to influence the HPA axis. Despite numerous studies reporting alteration of HPA axis signaling by sex steroids, their exact effect is still inconclusive. In animals, ovariectomy which removes endogenous estrogens in females reduces the HPA response to stimulation or stress, while supplementation of estradiol increases and restores the response (115–118). However, other studies reported an inhibitory effect (119, 120). This discrepancy could be due to dose and duration of treatment, as well as model differences and health status of the animals (121). In humans, while a study reported that estradiol enhanced the HPA response in men (122), studies in postmenopausal women reported the opposite response (123). In addition to the confounding factors referenced above, the type of receptors involved also influence the effect of estrogens. Activation of ERα exerts stimulatory effects on the basal state and during restraint stress, whereas activation of ERβ inhibits the HPA-axis only when this is activated (117, 124).

In contrast to estrogens, androgens generally inhibit HPA axis activity. Castration in male animals enhances HPA axis activity, while androgen treatment inhibits the activity (117, 118, 125, 126). The same effect of androgen supplementation are found in humans (127). In male mice with dysfunctional AR signaling, the HPA axis response to stress is elevated (128, 129), demonstrating that AR is involved in HPA axis suppression. DHT, a strong AR ligand, is often used to examine the effect of androgens and is shown to inhibit the HPA axis. As DHT cannot be converted into estradiol, its effect is not likely due to aromatization. Conversely, DHT can be metabolized into Androstan-3,17-diol (3β-diol) which binds to ERβ and could exert an inhibitory effect on the HPA axis. Thus, the conversion of DHT into 3β-diol and ERβ activation could mediate some of the effects of DHT (117).

### Autonomic Nervous System

In addition to the HPA axis, the autonomic nervous system is also a major component of the stress response. It consists of the sympathetic nervous system and parasympathetic nervous systems. While the sympathetic nervous system prepares the body for the ‘fight or flight’ response, the parasympathetic system restores the body to a relaxed state. The autonomic nervous system regulates body functions such as arterial pressure and heart rate, as well as numerous gastrointestinal functions including blood flow, peptide hormone release, visceral sensitivity and gastrointestinal motility. The autonomic system is reported to be disturbed in IBS. In general, sympathetic activity is upregulated while parasympathetic signals are downregulated in IBS (130), but the alterations could be dependent on IBS type and sex. Generally, males have higher sympathetic activity, while females have higher parasympathetic activity. Some studies focusing on females reported elevated sympathetic signaling with inhibition of the parasympathetic activity in IBS (131, 132). In another female IBS study, autonomic function was similar in IBS patients and healthy controls; while parasympathetic activity was lower in IBS-C than IBS-D patients (133). A separate study found that autonomic imbalance in IBS preferentially occurs in males (130).

Not surprisingly, the sexual dimorphic findings in autonomic neuronal function relate to sex hormones modulating this system. For instance, sympathetic activity is often increased in the luteal phase of the menstrual cycle or during menopause when estrogen levels are reduced (134). A study reported that surgical-induced menopause reduced parasympathetic nervous system activity and shifted this towards sympathetic hyperactivity (135). Although some studies found no effect (136, 137), others reported that estrogens in hormone replacement therapy facilitate parasympathetic activity and suppress sympathetic signaling in postmenopausal women (138–140). Estrogen may reduce sympathetic fiber density directly through affecting ERα expressed in sympathetic neurons, or indirectly through affecting target tissue or specific molecules (141). However, another study reported that estrogen is positively correlated to sympathetic activity in men (142). Nevertheless, estrogens are generally reported to inhibit sympathetic activity while sex could possibly influence the effect.

In contrast to estrogens, androgens are associated with sympathetic hyperactivity in females. In polycystic ovary syndrome patients, who often suffer from hyperandrogenism, sympathetic activity was enhanced whereas parasympathetic signaling was suppressed (143). Furthermore, excess neonatal androgen in female mice increases sympathetic tone in cardiometabolic tissues (144). However, several studies reported that androgens are positively correlated with parasympathetic activity in males (142, 145). Also, a study found that males with low testosterone levels were unable to maintain cardiosympathetic and cardiovagal responses (146). These inconsistent findings suggest that autonomic control mediated by sex steroids could be sex-dependent, as well as modulated by health and hormonal status of the individual.

### Enteric Nervous System

The enteric nervous system is often referred to as the “little brain” or ‘brain-in-the-gut’ and is the largest division of the peripheral nervous system. It is also considered as the third division of the autonomic nervous system. A distinguishing feature is it can act independently of the brain, but usually communicates with the CNS for regulation of enteric function and information transmission to the brain. The enteric nervous system is generally organized into submucosal and myenteric plexi that connect to the gut lumen *via* enteroendocrine cells in the epithelial layer and to the brain by vagal, spinal and sacral afferent nerves (147). The enteric nervous system regulates secretion and absorptive capacity in the intestine, as well as motility. In a rodent IBS model, animals exposed to stress exhibited more secreto-motor neurons in the submucosal plexus and less inhibitory musculo-motor neurons in myenteric plexus (148). There are also sex-dependent differences in the enteric nervous system, for example distinct structural and functional characteristics of enteric neurons in pigs (149, 150). Numerous components related to enteric nervous system function are influenced by IBS and sex steroids, although these tend to be more subtle.

Serotonin (5-HT) is a neurotransmitter closely associated with central and enteric nervous system function. It represents a central regulator of diverse physiological activities, including sleep, mood and cognition. It also plays an important role in visceral sensitivity and gut motility (151). Although serotonin is produced by central and peripheral nervous systems, the primary source is from the gut enteroendocrine cells that also express chemosensory receptors and transmit sensory signals from the gut lumen to the nervous system. Upon local mucosal stimulation, enteroendocrine cells secrete 5-HT which activates 5-HT_3_ and 5-HT_4_ receptors on enteric neurons to facilitate motor and sensory responses (152). Serotonin levels are affected by sex and hormonal status associated with estrous cycle. Compared with females, males have higher 5-HT synthesis rates in the brain after acute tryptophan depletion (153). Female IBS patients show greater 5-HT synthesis in the brain compared with healthy females, while this difference was not evident in IBS males (154). The sex difference in 5-HT signaling pathways in IBS can also be seen in response to drugs. For instance, 5-HT_3_ receptor antagonists, including alosetron is more effective in females than males (96). Estrogens promote serotonin synthesis (155) and animals with an ovariectomy have reduced serotonergic neuronal numbers and expression of 5-HT related genes (156). It is believed that the drop in estradiol levels during menopause or after giving birth reduces serotonin activity and causes mood disorders (157). Estrogens also modulate the expression of the serotonin reuptake transporter (SERT) (158), which retrieves released serotonin into neurons and controls the amount of bioavailable circulating neurotransmitter. This effect was reported to be dependent on the drug exposure length (159). Furthermore, estrogens affect 5-HT receptor expression. For instance, estradiol upregulates the density and ligand binding to the 5HT2_A_ receptor in brain in postmenopausal women (160, 161). The 5HT2_A_ receptor enhances smooth muscle contraction (162), and its polymorphism is suggested as a risk factor in IBS (163). Regarding the effect of androgens, a higher testosterone level is associated with higher serotonin tone in healthy men (164). Androgens upregulate 5-HT synthesis and SERT expression and ligand binding (165, 166). At the same time, serotonin modulates androgen reaction by suppressing AR activity (167).

Another important cellular component that is closely related to enteric nervous system differences in IBS are mast cells, which serve as neuroimmune effectors in the intestine. After activation, mast cells secrete mediators such as histamine and protease. These mediators signal to enteric neurons and induce visceral hypersensitivity or alter intestinal muscle contraction. In IBS, mast cell abundance is correlated with clinical symptoms including abdominal pain, bloating, and depression (168, 169) (170). Mast cell activity is also influenced by gender and sex steroids. In rodents, female mast cells release more histamine than male-derived cells in response to sex hormones (171). Also, female mice have higher mast cell capacity to synthesize and store mediators such as histamine, as well as experience greater intestinal permeability and serum histamine responses to restraint stress (172). In terms of hormonal effect, mast cells express estrogen receptors and are activated by estrogens. A previous study reported that estradiol binds to ER-α on mast cells and triggers activation *via* enhancing influx of extracellular Ca2+ (173). This effect is likely non-genomic due to the rapid onset of the response. Various studies have also demonstrated that estrogens can enhance mast cell numbers and their neuroimmune-targeted secretions (171–177). In contrast to estrogens, androgens appear to inhibit mast cell activity. A recent article reported that perinatal androgens reduce mast cell secretion and impart protection in mast-cell associated disease (178). Another study reported that exposure of human skin mast cells to testosterone reduced pro-inflammatory cytokine production, reflecting an anti-inflammatory response (179).

## Gut Microbiota

In recent years the notion of the gut-brain axis has been extended to include the microbiome, termed the ‘microbiota-gut-brain axis’ (180). The gastrointestinal tract harbors trillions of bacteria that signal *via* the gut-brain axis through many diverse interactions including serotonin release (181, 182). The gut microbiota play an important role in host health, and alterations in composition and function could contribute to IBS pathogenesis and therapeutic efficacy (183). Despite a lack of consensus on distinct microbial differences in IBS, intestinal microbiome features such as community richness has been proposed to correlate with severity of IBS symptoms (184, 185), but these findings are more often than not generalizable. Not only does the microbiota influence gut development and function, but it can also exert impact on extra-intestinal symptoms including depression (186). Importantly, it is highly likely that gut microbiota composition is influenced by IBS symptoms, particularly bowel movement which affect time and metabolic products that result from the interaction between the gut microbiota, host and dietary components.

Gut microbiota composition is closely associated with sex steroid levels in a reciprocal manner. In humans, systemic estradiol and testosterone are correlated with gut microbial diversity and profile in males and females (187, 188). The gut microbiota plays a pivotal role in regulating steroid metabolism, containing enzymes such as sulfatases and glucuronidases that deconjugate steroids for reabsorption in the intestine (189). Germ-free mice have high levels of fecal conjugated steroids (190), which is also observed after antibiotic use in humans, for example ampicillin use during pregnancy (191). Additionally, the gut microbiota degrade steroids into other metabolic products. Some bacteria influence the potency of estrogens by converting estrone into a more potent form estradiol and vice versa (192) (193). Specific bacteria e.g. *Steroidobacter denitrificans* and *Comamonas testosteroni* transform and utilize sex steroids (194, 195).

In addition to the modulation of steroid metabolism by microbes, sex steroids may also regulate gut microbiota community structure and function. Males and females exhibit different microbial profiles in animals and humans (196, 197). For example, *Akkermansia* is reported to be more abundant in females than in males (198, 199). Perturbations of sex hormones after ovariectomy or administration of sex steroids to animals shifts their gut microbiota profiles (200, 201). Steroids may also play a role in changing microbiome profiles during pregnancy and after menopause (202, 203), although several other factors undoubtedly influence these microbiota communities. Sex steroids influence gut microbiota by altering intestinal adaptive immune responses, as well as bacterial function. For instance, estrogens enhance the level of secretory immunoglobulin A (IgA) that binds to and controls bacterial growth (204). Conjugated estrogen and bazedoxifen reduce fecal β-glucuronidase (GUS) enzyme activity, which is involved in microbial deconjugation of steroids (205).

In view of the interplay between sex steroids and gut microbiota, bacteria could play an important role in mediating the effects of steroids and contribute to sexual dimorphism in IBS. This possibility is supported by the example of type 1 diabetes. As reported by a study in rodents, the female-predominant incidence of type 1 diabetes is dependent on microbiota (206). Serum testosterone is higher in germ-free female mice than in SPF females, while the opposite is evident in males. Transfaunation of male cecal content into female weanlings not only alters the recipients’ microbiome, but also increases their testosterone level and protects them from type 1 diabetes and autoimmune disease. Another study in mice demonstrated that the microbiota is closely associated with sex-bias in type 1 diabetes (196). Apart from type 1 diabetes, modulation of gut microbiota profiles by sex steroids also mediates their impact on metabolic syndrome (207). These studies provide insight for future studies to evaluate the association between sex steroids and the gut microbiota in IBS pathophysiology.

## Epigenetic Modulation of the Microbiome-Gut-Brain-Axis in IBS

IBS is a complex and multifactorial gut-brain axis disorder and epigenetics is believed to be one of the key mechanisms that links environmental factors to genetics in IBS. Epigenetics is the study of heritable changes of gene expression which are reversible and do not alter DNA sequence. The changes often involve mechanisms including DNA methylation, posttranslational histone modification and non-coding RNA. In an epigenetic model of IBS (208), it is proposed that early adverse life events result in epigenetic changes of the HPA-axis that subsequently alter responses to environmental stressor and enhanced cortisol production. Altered cytokine responses and 5-HT signaling pathways are affected and could contribute to the manifestation of IBS symptoms. In humans, genome-wide DNA methylation profiling of peripheral blood mononuclear cells of IBS patients identified various genes with altered DNA methylation status (209). In animal stress-induced IBS models, differentially methylated or expressed genes were identified, demonstrating the influence of stress on the epigenome (210). Furthermore, alteration of epigenetics by histone deacetylase inhibitor reduced visceral sensitivity in stressed animals (211). In our own studies, we have demonstrated that intestinal barrier dysfunction, is associated with epigenetic signals to early life stressors, and these mechanisms could be linked to intestinal inflammation and abdominal pain (212, 213).

Epigenetic changes are evident in response to various factors including dietary intake, physical activity and drugs. These factors are also closely associated with the gut microbiome, while the microbial metabolites influence epigenetics and drive host-microbial interaction. The gut microbiome mediates the effects of several environmental factors on enteric neurotransmission *via* epigenetic regulation (214). Low FODMAP diet, a low fermentable carbohydrates diet which improves IBS symptoms, modulates gut microbiota composition and reduces SCFAs which are recognized as histone deacetylase inhibitors and affect epigenetic processes. In a mouse study, gut microbiota regulated global histone acetylation and methylation, while the effect is disturbed by western diet consumption and is recapitulated by SCFAs supplementation (215). In another study, the SCFA butyrate contribute to the epigenetic effect on differentiation of colonic regulatory T cells (216). Other studies also demonstrated SCFA effects on histone modification in colon and brain in rodents (217, 218). These reports demonstrate that SCFAs are microbial products that can modulate epigenetic signals in IBS, possibly *via* altered microbiome-gut-brain axis signaling.

Sex steroids are also important epigenetic modulators. The crosstalk between the epigenome, sex steroids and their receptors is proposed as a mechanism for sexual dimorphism of nervous system and immune function. Estrogens and ER serve as epigenetic modulators through different mechanisms including alteration of DNA methylation (219) and histone modifications (220). These signals can influence various sex-dependent phenotypes, including enzyme expression, cognition and reproductive function (221). At the same time, epigenetic effects on ER result in changes in its gene expression and response to estrogen (222), as well as host phenotype including endocrine-resistance in breast cancer (223). Similar to estrogens, androgens and AR also possess epigenetic modulating effects. These signals are reported to be critical in polycystic ovary syndrome, in which hyperandrogenism is the primary feature (224, 225). Furthermore, epigenetic regulation of AR by DNA methylation and histone acetylation was suggested to be associated with endocrine disorder and prostate cancer (226, 227). Several studies have focused on association of early androgen exposure and the epigenome during neuronal developmental stages. Perinatal testosterone modulates histone acetylation during brain development in mice (228). Disruption of epigenetic modulation alters the masculinizing effect of testosterone on sexually dimorphic brain structure development in mice (229). Taken together, sex steroids and gut microbiota modulate host phenotype and the gut-brain axis through epigenetic mechanisms. Future studies investigating how epigenetics are involved in the interplay between microbiota and sex steroids in IBS are needed.

## Conclusion and Future Perspectives

Sex steroids are closely associated with IBS onset and symptoms in multiple aspects. However, results regarding their exact role in IBS and underlying mechanisms have been inconsistent. At present, it is not clear whether an increase in sex steroids is beneficial or causative in IBS. This is partly explained by the complicated nature of steroid chemistry and their mechanism of action. Inconsistencies are also reflected in clinical cohort design and animal models used for study interpretation, emphasizing the need to stratify trial design by sex and standardizing treatment dose when studying sex steroids. Furthermore, whether gut microbiota can mediate the effects of sex steroids on IBS is still unclear and warrants further investigation as a precedent has already been set in other diseases. There is also little information about how adrenal androgens and the metabolic products of sex steroids are associated with IBS and gut-brain-axis signaling. This review provides a basis to continue the exploration of the complex interplay between sex steroids and metabolites, their receptors and the microbiome-gut-brain axis in the pathophysiology of IBS.

## Author Contributions

All authors contributed to the article and approved the submitted version. SS drafted the manuscript and created the figures, TS edited and finalized the manuscript.

## Funding

This work was supported by NIH grants from NIDDK P30-DK56338, NIAID R01-AI10091401, U01-AI24290 and P01-AI152999, and NINR R01-NR013497.

## Conflict of Interest

The authors declare that the research was conducted in the absence of any commercial or financial relationships that could be construed as a potential conflict of interest.
